# Large axial field of view PET/CT – Not just a longer PET gantry^☆^

**DOI:** 10.1016/j.zemedi.2026.03.008

**Published:** 2026-03-25

**Authors:** Mathias R. Steffner, Tim Felgenhauer, Bastian Szermerski, Johanna Diekmann, Frank M. Bengel, Sibylle I. Ziegler, Lilli Geworski

**Affiliations:** a Department of Radiation Protection and Medical Physics, Hannover Medical School, Germany; b Department of Nuclear Medicine, Hannover Medical School, Germany

**Keywords:** LAFOV PET/CT, Total-body PET/CT, Nuclear medicine, Positron emission tomography

## Abstract

Positron Emission Tomography (PET) has undergone disruptive advancements in the past two decades. One of the most impactful technological developments is the introduction of Large Axial Field-of-View PET/CT (LAFOV PET/CT). These systems are transforming clinical routine by enabling dramatically shorter acquisition times and substantially higher sensitivity, thereby influencing all related workflows. Established procedures gain new diagnostic potential thanks to image quality and detection accuracy that surpass the limits of conventional PET/CT. Beyond routine clinical applications, advanced methods such as parametric imaging or system-based network analyses particularly benefit from LAFOV PET/CT, as the extended field of view allows for the simultaneous observation of all relevant organs in a single bed position. This review highlights the main system features of LAFOV PET/CT and their potential in clinical use.

## Introduction

Positron emission tomography (PET) has been an established procedure in nuclear medicine for many years. Recent technological advancements have led to the availability of PET/CT hybrid systems with large axial fields of view, which enable total-body imaging in a single bed position with concurrently increased sensitivity. These features provide two fundamental possibilities for the further development of clinical applications. A long axial field of view allows for both static and dynamic data acquisition across large regions of the body, which is particularly useful in the assessment of tracer biokinetics. The improved sensitivity can also be used to reduce the examination time and thus increase patient throughput. In addition, the burden is reduced on patients who cannot tolerate the long scan times (20 – 30 min) in PET acquisitions. Furthermore, radiation exposure can be reduced significantly by lowering the amount of activity administered, which may be important especially in pediatric patients.

In recent years, PET has seen significant progress. Advancements in the radiopharmaceutical field, but also technological progress, have further increased the relevance of PET within nuclear medicine. In particular, in the field of whole-body imaging in nuclear medicine, PET/CT accounts for a significant part of all examinations in Germany [1].

The available hybrid systems primarily differ in the length of their axial field of view (FOV), which is reflected in a nomenclature that has been proposed to distinguish between different available types of PET/CT [2]:

1. PET/CT with short-axial field-of-view (SAFOV): Devices with a limited axial FOV typically cover 15 cm to 30 cm and a single organ of interest. Multiple bed positions are required for a scan of the total body.
2. PET/CT with long-axial field-of-view (LAFOV): Devices that enable simultaneous imaging from head to thigh in a single bed position. This nomenclature will be used in this work, despite Total-body is increasingly also used for LAFOV PET devices
3. Total-body (TB) PET/CT: Devices that allow imaging from head to toe in a single bed position.

Initial concepts towards LAFOV PET/CT systems were realized as early as 2003 [3], [4], [5], [6], and scanner configurations were investigated toward Total-body geometries in 2012 [7]. In 2019, the development of Total-body PET led to the first commercially available Total-body PET/CT system with an axial FOV of 194 cm, the uEXPLORER, developed by United Imaging Healthcare and the EXPLORER Consortium between UC Davis and the University of Pennsylvania [8], [9], [10]. A considerable benefit of this new generation of systems is the substantially increased sensitivity [11], [12]. Moreover, covering the entire body or all relevant organs in one bed position allows for entirely new imaging approaches to observe physiological processes [11], [13]. These benefits come – partly in the most literal sense – at a price. Especially in existing facilities, the significantly increased space requirements pose an impediment to the introduction of Total-body PET, at least without structural modifications [14]. The greatest obstacle to acquiring a Total-body PET system, however, are likely to be the higher acquisition costs, which, depending on the local reimbursement policies, may be difficult to amortize even with increased patient throughput [14]. With a focus on very high patient throughput LAFOV PET/CT nevertheless can be operated cost effectively [15]. Shorter LAFOV PET/CT systems offer a compromise between axial PET FOV and sensitivity with respect to acquisition costs and required floor space. The currently available and announced LAFOV systems offer axial PET FOVs of 106 cm, 128 cm, and 148 cm. The market thus offers a range of solutions – from the maximum technically feasible to space-saving systems at the lower end of the LAFOV spectrum – allowing clinical users to select according to their needs (Table 1).

**Table 1:** Overview of devices available with published performance parameters according to NEMA NU 2-2018

Parameter	Biograph Vision Quadra (Siemens Healthineers, Erlangen) [16]	uExplorer (United Imaging Healthcare, Shanghai) [17]	uMI Panorama GS (United Imaging Healthcare, Shanghai) [18]
Axial FOV	106 cm	194 cm	148 cm
Crystal	LSO 3.2 x 3.2 x 20 mm^3^	LYSO 2.76 x 2.76 x 18.1 mm^3^	LYSO 2.76 x 2.76 x 18.1 mm^3^
Sensitivity (70 cm line source; 0 cm radial offset)	175.3 kcps/MBq	174.0 kcps/MBq	176.3 kcps/MBq
TOF resolution (@ 5.3 kBq/ml)	225 ps	505 ps	188.8 ps

The devices listed are equipped with detectors using lutetium oxyorthosilicate (LSO) or lutetium-yttrium oxyorthosilicate crystals (LYSO) which are capable of supporting high resolution Time-of-Flight (TOF) technology. TOF technology was introduced into clinical PET systems as early as the 1980s [19], [20], [21], staying in use for more than 10 years [22]. By localization of the origin of annihilation events to a segment along the line of response (LOR), not only was the visual impression of the image improved, but also the Contrast Recovery Coefficient (CRC) [19]. Effective sensitivity increases inversely proportional to TOF timing resolution [23]. The effective sensitivity gain from TOF also is highest for LORs that traverse long distances, as is the case for PET systems with long axial fields of view [6], [19], [24]. Further technical improvements were achieved with the introduction of silicon photomultipliers (SiPM), which further enhanced the temporal resolution of TOF [6], [25]. SiPMs also allowed for higher spatial resolution as well as full crystal coverage [26].

GE Healthcare offers the Omnis Legend 128, a LAFOV PET/CT with a 128 cm AFOV based on bismuth germanium oxide (BGO) crystal detectors. Since until publishing this article no performance paper was available, it is thus not listed in Table 1.

Compared to the bismuth germanium oxide crystals (BGO) which provide a higher detector sensitivity, LYSO has a lower effective atomic number but surpasses BGO in terms of light output, temporal resolution, and energy resolution [27]. The intrinsic activity of Lu-176 in the crystals can also be utilized for quality assurance and consistency checks [28] and attenuation correction [29] but might be considered for data acquisition with very low count rates such as Y-90 [30].

## Sensitivity, Dose Reduction, and Patient Throughput

Early PET systems using 2D acquisition could only detect coincidence lines (LOR) from within the X-Y-plane of each detector ring [31]. This meant that only annihilations occurring in the transverse plane of each detector ring could be detected. The geometry of the measurement arrangement is of great importance here. From a point source located centrally in one detector ring, observation of the omnidirectionally emitted LORs would require a spherical detector. For instance, assuming a single detector ring width of 1 cm and a detector ring diameter of 70 cm, theoretically, assuming 100% efficient detector elements, fewer than 2% of the LORs would be measurable. With the block detector concept [32] even early PET systems consisted of several axially stacked detector rings to increase sensitivity.

3D PET was developed and became available in the early to mid-90s. In contrast to the 2D PET described above, this generation of devices did not use septa between the individual detector rings. This made it possible to detect coincidence lines across the entire axial detector width and several detector rings, necessitating reconstruction algorithms including oblique angles [33]. While sensitivity is higher in 3D PET, the number of detected scatter events is also increased. Thus, improved scatter correction algorithms for 3D PET are essential for quantitative PET [34]. To date, 3D PET technology is standard in commercially available PET/CT systems. Geometry, in turn, plays a major role in the increased sensitivity of 3D PET. While 2D PET can only detect coincidence lines that are almost completely orthogonal to the Z-axis of the system, the geometric acceptance of 3D PET technology can be extended to the entire detector length in the Z-axis. However, the sensitivity along the Z-axis is not constant, as coincidences at the outer ends of the tomograph can only be detected orthogonally to the Z-axis, since any larger angle of the coincidence lines to the Z-axis would result in at least one photon of the annihilation being emitted toward outside the detector. The sensitivity profile is therefore minimal at both ends of the detector and increases towards the center of the scanner.

For 3D PET technology, the length of the system in the Z-axis is therefore decisive for the sensitivity of the overall system. However, the increasing sensitivity is only applicable to a limited extent, especially for very long axial FOVs. If the axial FOV becomes too long, or the maximum angles of the LOR relative to the transverse plane become too large, coincidence detection becomes increasingly difficult. For LORs traversing long distances through tissue, as it is the case at these large angles, attenuation becomes more prominent. Hence more random and scattered photons are detected in relation to true incidents [11]. To compensate for this, more sophisticated correction methods are needed or the small reduction in sensitivity is accepted by restricting the angle [11]. Moreover, parallax errors arise due to the radial length of the detector crystals, which lead to a decrease in spatial resolution, primarily axial [35], [36], [37], [38]. To reduce these challenges, the principle of maximum ring difference (MRD) can be applied. This limits the axial acceptance range, i.e., the axial length over which coincidences are detected. Equivalently, the MRD can be described as the maximum axial acceptance angle (MAA). However, this limitation reduces the effective sensitivity and restricts the axial sensitivity profile, transforming the triangular profile into a trapezoid.

Schmidt et al. examined the influence of MRDs of different lengths for the Siemens Vision Quadra with the introduction of the Ultra High Sensitivity (UHS) mode and found a degradation in axial resolution compared to the High Sensitivity (HS) mode [37]. Spatial resolution in radial and tangential axis however remained similar [37]. The MAA for HS mode is 18° and for UHS mode 52°, with LORs being used over the entire detector length for UHS mode [37]. In UHS mode, a triangular sensitivity profile can therefore be expected. LAFOV PET from United Imaging has an MAA of 57° with the uExplorer [17] and an MAA of 62° with the Panorama GS [18]. The Panorama GS also uses the entire detector length of 148 cm to detect coincidences. The axial sensitivity profile is therefore also approximately triangular, with maximum sensitivity in the center of the axial FOV.

The increased sensitivity offers a number of clinically useful advantages. For example, dosimetric measurements can be performed at later time points than previously possible. To validate proper application after intra-arterial selective internal radiotherapy (SIRT) with Y-90, imaging of bremsstrahlung can be performed using SPECT or of internal pair production using PET [39]. Since this transition is a highly unlikely process, a PET system with higher sensitivity potentially offers advantages in imaging and thus also in post-therapeutic dosimetry [39].

A reduction in the required activity, in turn, offers new opportunities to achieve a balance between radiation exposure and achieved image quality [40]. A low required activity also means that, for example, a Ge-68/Ga-68 generator can be in use for longer, or that imaging is still possible even with low chemical labeling yields [41]. Reducing the activity can also offer economic savings potential for commercially purchased radiopharmaceuticals such as F-18-FDG. Not only but specifically in pediatric patients [42], acceptable image quality can be achieved with reduced activities, thus also reducing the exposure associated with the procedure. Using reduced activities can also be applied on longer lived PET nuclides and nuclides with lower branching ratio for positron emission such as Zr-89 and Cu-64 whilst achieving a reasonable dose exposure. Systems offer the option of replacing the attenuation CT with an AI-based “CT”, which can further reduce exposure for hybrid imaging [43]. Finally, yet importantly, small amounts of activity lead to potentially lower radiation exposure for staff.

In addition to the possibility of dose reduction, the acquisition time can also be reduced. In particular, a shorter acquisition time is helpful for patients who cannot tolerate long periods of scanning. This can be due to pain or claustrophobia. For patients who need anesthesia, short scan times are also advantageous.

## Parametric Imaging, Radiomics and System-Based Network Analyses

Biokinetic modeling in the case of e.g. FDG-PET/CT covering quantities like glucose transport and metabolic rate requires additional input. This includes an arterial blood input function (BIF) and the dynamic scan of the tracer distribution, enabling an estimation of time-activity curves (TACs) in regions and organs of interest. Using a SAFOV-PET, the required TACs and BIF come with a compromise: The dynamic scan can only be acquired over the small FOV of maximum 30 cm. In order to acquire more organs with one scan, the patient table needs to pass through the PET gantry several times to obtain TACs for the whole body. This comes at the cost of sacrificing temporal resolution and loss of counts due to parts of the body being temporally out of the axial FOV [44], [45].

The LAFOV PET has the advantage that it may observe an area enabling measurement of a BIF (e.g. aorta) while simultaneously acquiring the body from head to thigh. The large FOV results in more consistent images with the patient staying in one bed position and the possibility of high quality direct parametric reconstruction, which includes the Patlak model on a voxel-wise level [46].

Apart from Patlak analysis, other, non-linear modeling approaches for different PET Tracers are also of interest; alongside the continuous development of novel radiotracers, studying them on a dynamic level is important, since a static scan oftentimes simplifies potentially relevant information of radiopharmaceutical distribution [13]. The biologically relevant parameters gained from parametric imaging may become important tissue characterization not only in tumors, but also in other situations, such as inflammation (see below).

The aim of radio(gen)omics is to help in patient risk stratification and prognostic modeling. Observing the biokinetics of the entire patient in one bed position could promise new opportunities for radiomics, especially using dynamic scans with high temporal resolution. Thus it has a demand for extensive data analysis and interdisciplinary collaboration. Systematic reviews show growing interest in the analysis of especially lung cancers, but also throughout other oncological areas [47], [48]. Even though radiomics showed constraints with regard to reproducibility [49]. LAFOV PET-based analysis showed promising results, as it provides higher sensitivity and better signal-to-noise-ratio allowing for higher reproducibility in image acquisition [50].

Ultimately, in total-body imaging, the concept of radiomics can be taken to a different scale level, where analysis goes beyond a certain region of interest and includes the entire organism. Whole-body features may then be analyzed to identify connections between various tissues and organs. This requires automated postprocessing tools that enable the robust segmentation of body regions from CT data, their transfer to the co-registered PET data, and the subsequent detection of similarities or dissimilarities in signal patterns to detect connections between body regions in groups of various disease states and to ultimately describe the individual phenotype with advanced, systems-based precision [51], [52], [53].

## Clinical applications of LAFOV PET/CT

LAFOV PET/CT scanners combine ultrahigh sensitivity, extended anatomical coverage, and improved spatial and temporal resolution, enabling novel diagnostic and therapeutic applications across a broad range of clinical settings. Their capabilities include streamlined whole-body dynamic imaging, multiparametric analyses, and reliable quantification at lower tracer activities, which collectively support the shift toward more personalized and system-oriented medicine [54], [55].

### Oncology

In oncology, enhanced spatial resolution and sensitivity are particularly relevant for lesion detection and quantification. Even small or low-uptake lesions that may be overlooked on conventional scanners can be detected more reliably with LAFOV PET/CT. In a large retrospective study, nearly one in twenty patients undergoing F-18-FDG PET/CT for staging had lesions detectable only due to the increased sensitivity of LAFOV imaging, with melanoma, breast cancer, and multiple myeloma patients benefiting most [56].

LAFOV PET also supports advanced multiparametric imaging of tumor biology. For instance, perfusion imaging with Rb-82 combined with FDG-PET metabolic assessment allows characterization of perfusion–metabolism mismatches, a marker of tumor aggressiveness and vascular inefficiency [57]. Furthermore, advanced modeling with high-temporal-resolution LAFOV PET now enables perfusion imaging with a wide range of tracers, including FDG, which may substantially facilitate perfusion–metabolism mismatch studies in the future[58]. In addition, dual-tracer protocols become feasible within a single day. A study of neuroendocrine neoplasms demonstrated that low-dose F-18-FDG followed by F-18-SiFAlin-TATE PET provided clinically reliable quantification while influencing therapy decisions in over half of patients, thus reducing logistical burden and radiation exposure [59].

Ultralow-dose protocols further extend applicability. In oncology patients, quantitative parameters such as SUVmean and SUVpeak remained robust even at 0.3 MBq/kg FDG, supporting reliable lesion detection while keeping radiation exposure as low as reasonably achievable [60]. These features are especially relevant in radiation-sensitive populations, including young adults and pregnant women.

### Pediatrics

Children particularly benefit from LAFOV PET/CT due to the ability to combine high-quality imaging with reduced radiotracer doses and shorter acquisition times. Half-dose F-18-FDG PET/CT has been shown to maintain diagnostic accuracy at scan durations as short as 60 seconds [61]. Critically, these rapid and low-dose protocols minimize or eliminate the need for anesthesia, which carries considerable risks in pediatric populations. This not only improves patient safety but also facilitates repeated follow-up imaging over the course of treatment.

### Inflammation and musculoskeletal disorders

Conventional PET imaging has long faced limitations in inflammatory diseases, including suboptimal spatial resolution and dose concerns. LAFOV PET/CT enables high-quality imaging of systemic inflammation and local lesions with extended uptake times or dynamic acquisition [62].

In musculoskeletal disorders, total-body coverage allows simultaneous assessment of the axial and appendicular skeleton. This has implications for diagnosis and management of inflammatory arthritis, osteoarthritis, infections, and metabolic bone diseases such as osteoporosis [63], [64].

High-resolution vascular imaging has also emerged as a strength of LAFOV technology. For example, vasculitic involvement of medium-sized vessels, which was previously beyond the scope of conventional PET, can now be detected with high accuracy [65]. Such detailed vascular assessments may significantly improve outcomes through earlier diagnosis and treatment initiation.

### Cardiovascular Imaging

In cardiovascular disease, LAFOV PET/CT facilitates detailed vessel wall and myocardial imaging. Vessel wall uptake of FDG can be assessed over prolonged time windows or at ultralow tracer doses, supporting safe longitudinal studies in low-risk populations [66]. Furthermore, emerging evidence suggests that total-body perfusion imaging with LAFOV PET may enable quantitative assessment of microvascular function and peripheral vascular disease, potentially extending its applicability beyond large-vessel imaging to include systemic vascular evaluation [67].

Beyond morphology, multiparametric dynamic imaging provides novel insights into systemic and myocardial metabolism. LAFOV PET/CT allows simultaneous kinetic modeling across the entire body, enabling detailed characterization of myocardial glucose, fatty acid, and oxidative metabolism, as well as systemic organ crosstalk in conditions such as heart failure [68]. Tracers targeting fibrosis (e.g., FAPI) further expand applications, offering opportunities to study myocardial remodeling and its systemic impact [68].

### Radionuclide therapy and dosimetry

The integration of LAFOV PET/CT into therapeutic workflows is particularly promising. In radiopharmaceutical therapy (RPT), accurate and personalized dosimetry is critical but has historically been limited by scanner sensitivity. With LAFOV PET/CT, dynamic whole-body imaging allows more precise evaluation of tumor distribution, tracer kinetics, and heterogeneity, enabling personalized therapy schemes rather than “one-size-fits-all” protocols [54].

In Y-90 transarterial radioembolization, LAFOV PET demonstrated superior quantification of absorbed doses and improved noise characteristics compared with conventional PET, thereby enhancing tumor dosimetry [69]. However, further refinement of voxel-wise dosimetry methods is required to fully exploit the sensitivity of LAFOV scanners [70].

## LAFOV PET instead of Total-body PET – a step back or a necessary compromise?

**Fig. 1:**
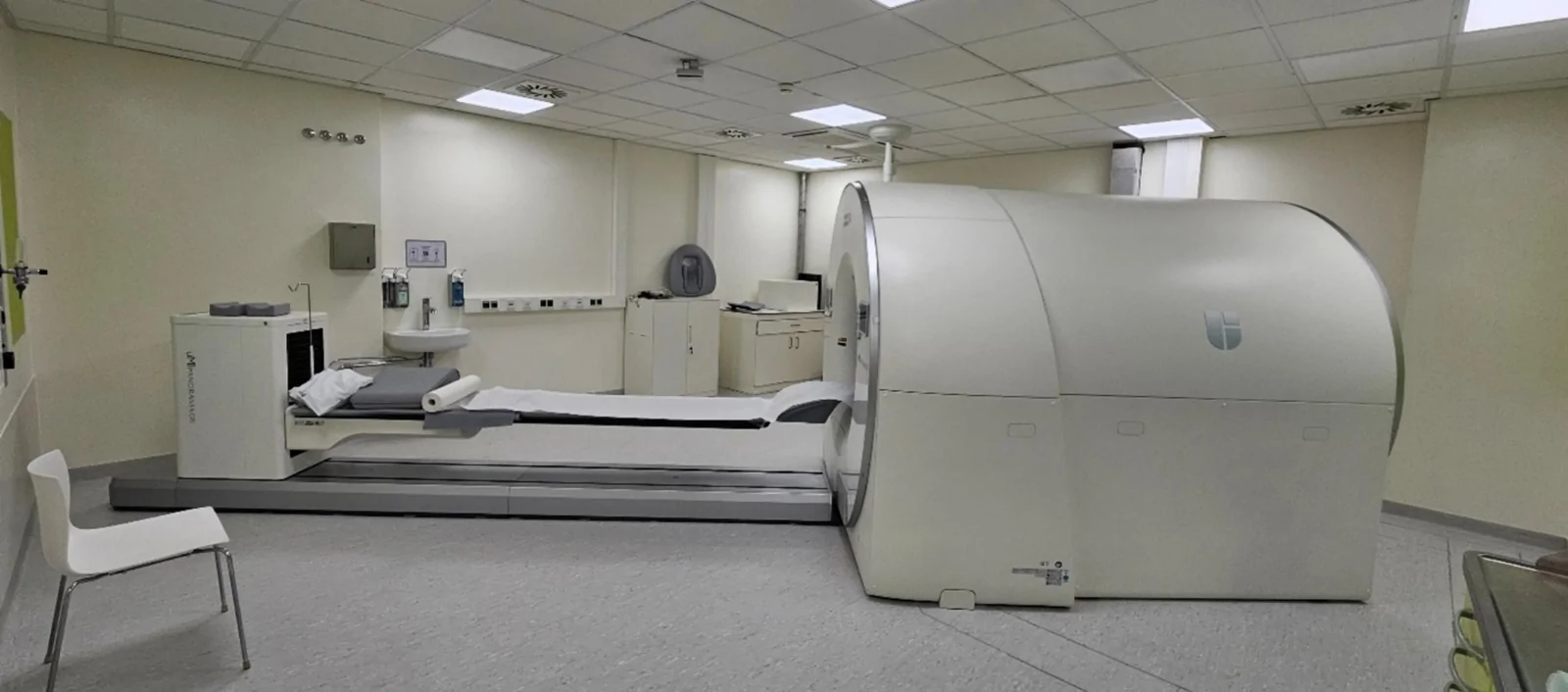
Photograph of United Imaging Healthcare - Panorama GS LAFOV PET/CT installed at Hannover Medical School

The space requirements for a hybrid system such as the TB or LAFOV version of a PET/CT need to be considered, as it may be difficult to adapt in existing buildings. For the currently shortest LAFOV PET/CT, the Siemens Biograph Vision Quadra, space requirements are comparable to those of conventional PET/CT systems [14], yet this comes at the expense of a 46 % shorter axial PET FOV compared to the longest available TB PET/CT [14]. Thus, axial PET FOV can be maximized within the given constraints. Fig. 1 shows an example of a 148 cm LAFOV PET/CT in an approx. 50 m^2^ room. An increased space requirement for technical equipment rooms cannot generally be assumed for either LAFOV or TB PET/CT systems.

The amount of data generated by LAFOV PET or even TB PET systems far exceeds that of SAFOV systems, especially with dynamic scans over the full length of the gantry. This is for example relevant in case of list-mode acquisitions which can generate up to 1 TB of data per hour [14]. Accordingly, greater computational power is necessary to reconstruct this data within the usual timeframe. While this data initially occurs only on the device side, the associated IT infrastructure must be able to handle long-term storage and further processing, requiring appropriately scaled capacity. Storing list-mode data in external archives, e.g. for retrospective reconstructions or analysis, can require petabyte-scale storage [14]. Owing to the large number of slices and matrix sizes, increased computational power might also be necessary for image viewing stations and archiving.

The procurement costs of the devices play a major role. Dadgar et al. estimated the manufacturing costs of a Total-body PET/CT [71]. The estimated primary cost factors are the detectors, i.e., scintillation crystals, light sensors, and readout electronics [71]. Reducing the axial PET FOV below the Total-body PET length is therefore an approach to reducing costs, but at the expense of the axial FOV. Despite the reduced axial FOV, shorter LAFOV PET/CT systems still offer significant advantages over SAFOV PET.

In addition to the acquisition costs, higher maintenance and operating costs may be the consequence of the much larger number of system components. However, higher patient throughput due to shorter scanning times can also offer economic advantages besides improved patient comfort. This should be considered in comparison to operating e.g. two SAFOV PET devices instead of one LAFOV PET, in case high patient throughput is the primary goal without focus on true simultaneous acquisition of different organs [15].

### Summary

Since the FDA clearance of the first Total-body PET/CT device in 2018 [17] adoption of LAFOV scanners has grown steadily, with over 50 installations worldwide [55]. Key advantages include:

• Enhanced lesion detection and quantification, improving oncology staging, therapy monitoring, and assessment of systemic inflammatory diseases [56], [63], [64], [65]
• Reduced dose and shortened acquisition times, particularly beneficial for pediatric and radiation-sensitive populations, reducing the need for anesthesia [60], [61], [72]
• Systems-based imaging, enabling simultaneous evaluation of cardiovascular, metabolic, and inflammatory processes across multiple organs [57], [66], [68]
• Improved therapy planning and dosimetry, paving the way for individualized RPT approaches [54], [69], [70]
• Feasibility of novel protocols, including dual-tracer imaging within a single day [59].

Despite challenges such as cost and data management, the clinical benefits of LAFOV PET/CT – particularly in oncology, pediatrics, inflammatory disease, cardiovascular medicine, and radionuclide therapy – position this technology as a cornerstone of precision imaging for the coming decades.

### Outlook

Developments in detector technology, electronics, and reconstruction algorithms including state-of-the-art computing have facilitated the design of LAFOV PET/CT devices which enhance clinical imaging and open new opportunities for radiotracer imaging research. It will be interesting to see how new solutions currently being investigated will perform in the clinical arena in the future. These include the use of plastic scintillation detectors [73] or LYSO/SiPM panel detectors for upright PET scanning [74]. While technology for total-body coverage is attracting a lot of attention, development of PET devices with non-standard detector geometry, for example organ-specific PET or walk-through PET, is ongoing [75]. These devices will greatly benefit from future technological advancements in TOF performance. For now, the growing availability of LAFOV and Total-body PET/CT technology in different configurations will surely advance its relevance in the field of nuclear medicine.

## CRediT authorship contribution statement

**Mathias R. Steffner:** Writing – original draft. **Tim Felgenhauer:** Writing – original draft. **Bastian Szermerski:** Writing – original draft. **Johanna Diekmann:** Writing – original draft. **Frank M. Bengel:** Writing – original draft. **Sibylle I. Ziegler:** Writing – original draft. **Lilli Geworski:** Writing – original draft.

## Declaration of competing interest

The authors declare that they have no known competing financial interests or personal relationships that could have appeared to influence the work reported in this paper.
